# The Utilization of the Acyl-CoA and the Involvement PDAT and DGAT in the Biosynthesis of Erucic Acid-Rich Triacylglycerols in Crambe Seed Oil

**DOI:** 10.1007/s11745-014-3886-7

**Published:** 2014-02-28

**Authors:** Tomasz Furmanek, Kamil Demski, Walentyna Banaś, Richard Haslam, Jonathan Napier, Sten Stymne, Antoni Banaś

**Affiliations:** 1 Institute of Biology and Environmental Protection, Pomeranian University in Słupsk, Arciszewskiego 22b, 76-200 Słupsk, Poland; 2 Intercollegiate Faculty of Biotechnology of University of Gdańsk and Medical University of Gdańsk, Kładki 24, 80-822 Gdańsk, Poland; 3 Institute of Biology, University of Natural Sciences and Humanities, Prusa 12, 08-110 Siedlce, Poland; 4 Biological Chemistry Department, Rothamsted Research, Harpenden, Herts AL5 2JQ UK; 5 Department of Plant Breeding, SLU, Alnarp, Sweden

**Keywords:** Crambe, PDAT, DGAT, Microsomal preparation, Triacylglycerol, Erucic acid, Lipids

## Abstract

The triacylglycerol of *Crambe abyssinica* seeds consist of 95 % very long chain (>18 carbon) fatty acids (86 % erucic acid; 22:1^∆13^) in the *sn*-1 and *sn*-3 positions. This would suggest that *C. abyssinica* triacylglycerols are not formed by the action of the phospholipid:diacylglycerol acyltransferase (PDAT), but are rather the results of acyl-CoA:diacylglycerol acyltransferase (DGAT) activity. However, measurements of PDAT and DGAT activities in microsomal membranes showed that *C. abyssinica* has significant PDAT activity, corresponding to about 10 % of the DGAT activity during periods of rapid seed oil accumulation. The specific activity of DGAT for erucoyl-CoA had doubled at 19 days after flowering compared to earlier developmental stages, and was, at that stage, the preferred acyl donor, whereas the activities for 16:0-CoA and 18:1-CoA remained constant. This indicates that an expression of an isoform of DGAT with high specificity for erucoyl-CoA is induced at the onset of rapid erucic acid and oil accumulation in the *C. abyssinica* seeds. Analysis of the composition of the acyl-CoA pool during different stages of seed development showed that the percentage of erucoyl groups in acyl-CoA was much higher than in complex lipids at all stages of seed development except in the desiccation phase. These results are in accordance with published results showing that the rate limiting step in erucic acid accumulation in *C. abyssinica* oil is the utilization of erucoyl-CoA by the acyltransferases in the glycerol-3-phosphate pathway.

**Electronic supplementary material:**

The online version of this article (doi:10.1007/s11745-014-3886-7) contains supplementary material, which is available to authorized users.

## Introduction

**Scheme 1:**
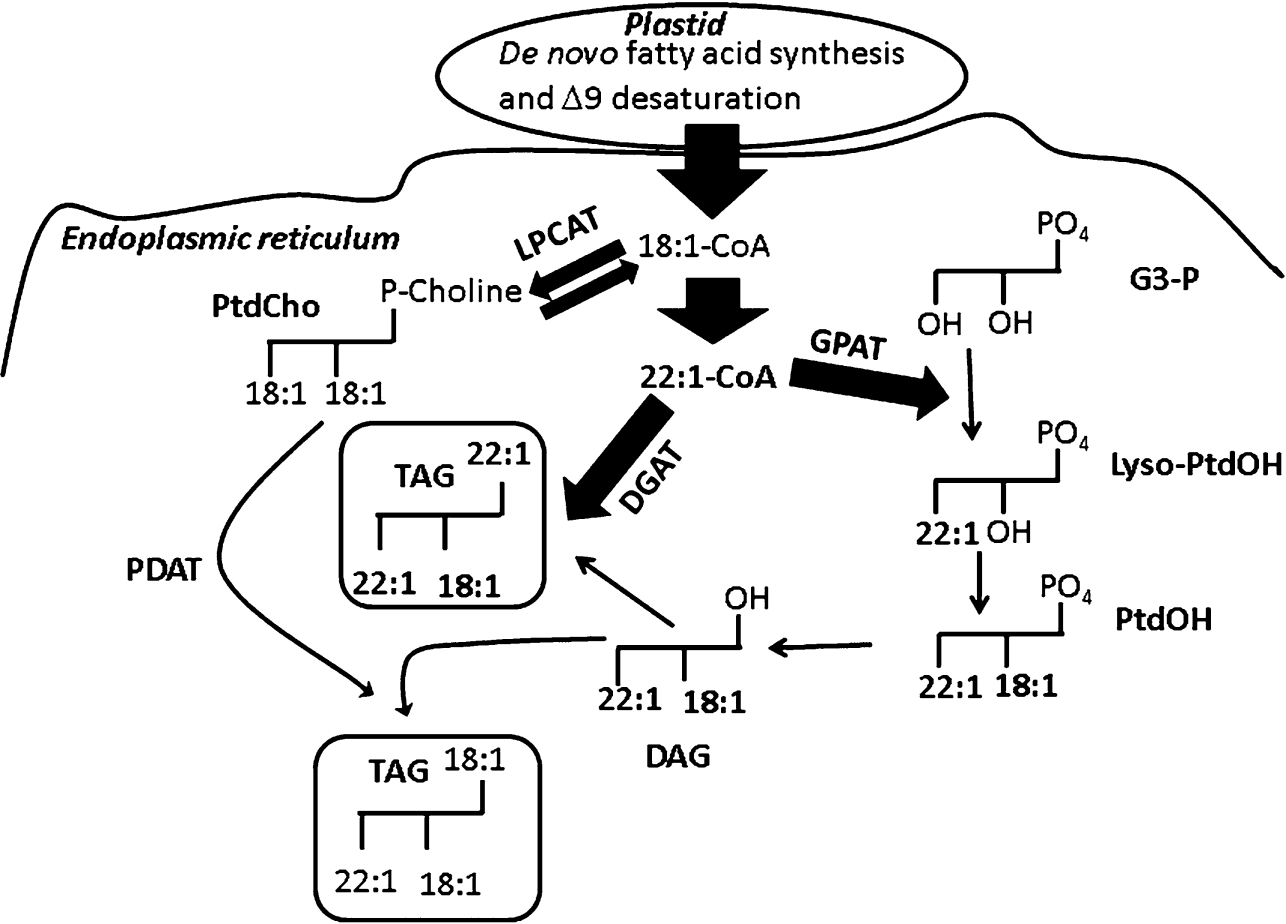
Proposed schematic flow of oleoyl (18:1) and erucoyl (22:1) acyl groups to triacylglycerols (TAG) in Crambe seed cells. The thickness of the *filled arrows* represents roughly the proportions of the various flows. Oleate produced in the plastid is exported out in the cytosol where enzymes in the endoplasmic reticulum catalyze the depicted reactions. Oleate from oleoyl-CoA enters phosphatidylcholine (PdtCho) reversibly via the acyl-CoA:lysophosphatidylcholine (LPCAT) catalyzed reaction. The main part of the oleoyl-CoA is elongated to erucoyl-CoA which is used by the acyl-CoA:glycerol-3-phosphate acyltransferase (GPAT) in the formation of lysophosphatidic acid (lysoPtdtOH) and the acyl-CoA:diacylglycerol acyltransferase (DGAT) in the formation of triacylglycerols. The oleoyl-CoA and other C18 acyl groups are esterified mainly at the *sn*-2 position of the glycerol backbone to form phosphatidic acid (PtdOH). Triacylglycerol formation also occurs via the phospholipid:diacylglycerol acyltransferase (PDAT). The DGAT catalyzed reaction results in TAG with mainly erucic acid in the outer positions and C18 fatty acids in the middle, *sn*-2 position whereas the (minor) PDAT catalyzed reaction results in TAG with erucic acid only at the *sn*-1 position

Crambe (*Crambe abyssinica* Hochst.) is an annual herbaceous plant belonging to the Brassicaceae family. It produces white inflorescences of raceme type and seeds in siliqua fruits that do not open when ripe. Cultivated commercially as an oil crop only on a small scale in a few countries, its oil contains up to 60 % of erucic acid (22:1^Δ13^) and is therefore used exclusively for industrial purposes. Processed 22:1 (the main product is erucamide), is used in production of plastics, nylon 13-13 and high temperature lubricants [1–3]. The low content of polyunsaturated fatty acids and enrichment of 22:1 found in Crambe oil offers greater potential to the chemical industry than the high erucic acid rape seed oil. Latterly, transgenic Crambe with over 70 % of 22:1 in its oil has been developed [4]. The distribution of 22:1 in non-transformed Crambe seed triacylglycerols (TAG) is uneven, with a very low amount in the middle, *sn*-2 position, and the majority (86 %) located at the *sn*-1 + *sn*-3 positions [4]. In addition to 22:1, other long chain fatty acids (>18 carbon) occupy these positions, resulting in nearly 95 % of very long chain fatty acids in the outer positions of TAG [4]. Despite the observed accumulation of 22:1 in TAG, Crambe has a low level (about 5 %) erucic acid in phosphatidylcholine (PtdCho), the major membrane lipid. De novo fatty acid synthesis in plant cells occur in the plastid. In most oil seeds, the major forms of fatty acids exported from the plastid is oleoyl-CoA (18:1-CoA), palmitoyl-CoA (16:0-CoA) and stearoyl-CoA (18:0-CoA) [5]. Erucic acid (22:1) is synthesized outside the plastid by the malonyl-CoA-dependent elongation of 18:1 catalyzed by the condensing enzyme fatty acid elongase (FAE or KCS), a reductase, a hydratase and an enoyl reductase. In oil seeds, the polyunsaturated fatty acids, linoleic (18:2) and linolenic (18:3) acids, are produced as precursor fatty acids are esterified to PtdCho [5]. These fatty acids are then transferred to TAG by three major pathways: (i) transferred from PtdCho into the acyl-CoA pool where they can be acylated at all three positions of the glycerol molecule by the glycerol-3-phosphate pathway [5]; (ii) enter the diacylglycerol (DAG) pool by interconversion of PtdCho with DAG synthesized by glycerol-3-phosphate pathway, a reaction catalyzed by phosphatidylcholine:diacylglycerol cholinephosphotransferase (PDCT) [6]. The PtdCho derived DAG could then be acylated by the diacylglcyerol acyltransferase (DGAT) to form TAG. In this case the distribution of fatty acids at the *sn*-1 and *sn*-2 positions of the newly formed TAG molecule will be the same as in the PtdCho molecule from which it was derived. And (iii) acyl groups from mainly position *sn*-2 of PtdCho can be acylated to the *sn*-3 position of DAG in the formation of TAG in a reaction catalyzed by the phospholipid:diacylglycerol acyltransferase (PDAT) enzyme [7]. In Arabidopsis, 40 % of the polyunsaturated fatty acids found in TAG are believed to be channeled via PtdCho-DAG interconversion [6]. The contribution of PDAT to TAG synthesis has not been established, but it is believed that it can be significant in some oil seeds that are high in polyunsaturated fatty acids like safflower [8] and linseed [9]. Crambe offers a unique example of an oil seed that has TAG with fatty acids at the *sn*-1 and *sn*-3 position that are nearly exclusively synthesized outside PtdCho and are at very low in levels in this lipid. It has recently been suggested that the very low PDCT activity in Crambe contributes to the exclusion of these very long chain fatty acids from entering PtdCho and C18 fatty acids to enter DAG via PtdCho-DAG interconversion [10]. It should be noted that most acyl groups are entering PtdCho via the activity of the acyl-CoA:lysophosphatidylcholine acyltransferase (LPCAT) [5] and thus low activity of this enzyme towards 22:1-CoA is also likely to contribute to the low levels of 22:1 in this lipid. The proposed relative flows of 22:1 and 18:1 acyl groups into TAG in Crambe seed cells are depicted in Scheme 1. In the work presented here, we investigated the activity of PDAT in a microsomal preparation of developing seeds from Crambe to evaluate if a lack of this enzyme activity could explain the near absence of PtdCho-derived acyl groups in the outer positions of seed TAG. We also compared the activity of PDAT with DGAT, the enzyme that can be expected to be the dominating TAG synthesizing enzyme in Crambe. These activities were then correlated with the accumulation of various lipids during seed development as well as measurements of the acyl-CoA composition and amounts at various stages of seed development. Collectively, the results show that Crambe seeds have significant PDAT activity and that the 22:1-CoA proportion relative to other acyl-CoAs is far higher than the proportion of 22:1 found in TAG. Furthermore, studies of DGAT activity indicate that an isoform of DGAT with high specificity for 22:1-CoA is induced of the onset of rapid 22:1 acid accumulation in the seeds. The data are discussed in relation to the rather unique fatty acid composition of the TAG molecules in Crambe oil.

## Materials and Methods

### Plant Material

Seeds of *C. abyssinica*, cv. Mayer were planted in peat-based soil and transferred to a growth chamber with 60 % relative humidity and a 16 h photoperiod (200 μmol radiation; a day temperature of 21 °C; a night temperature of 18 °C). After a few weeks, the plants started flowering. At specific stages following flowering, the developing fruits were harvested and seeds were separated manually from the surrounding siliqua. Based on the days after flowering (DAF) and morphological criteria, the harvested seeds were classified into one of the six stages of seed development, prior to further analyses (Supplement Table S1).

### Substrates

Radioactive fatty acids were obtained from Biotrend, Cologne, Germany. Di-18:1-DAG, di-6:0-DAG were purchased from Sigma and unlabelled fatty acids supplied by Larodan Fine Chemicals (Malmö, Sweden). The [^14^C]acyl-CoAs and acyl-CoAs were synthesized according to modified method described by Sanchez et al. [11]. Radioactive DAG (*rac*-*sn*-1,2-di-[^14^C]18:1-DAG) was obtained by partial lipase (*Rhizopus arrhizus*; Sigma-Aldrich, St. Louis, MO, USA) treatment of tri-[^14^C]18:1-TAG (Perkin Elmer, Waltham, MA, USA) followed by separation of the obtained lipid products using thin layer chromatography (TLC), elution from the gel and determination of concentration by analyzing the fatty acid content of aliquots as methyl esters on GC with methyl-heptadecanoic acid added as an internal standard as described below.

### Microsomal Preparation and Enzyme Assays

Microsomal membranes were prepared from freshly harvested seeds (separated from the surrounding siliqua). The seeds coats were removed manually and microsomes were prepared according to the method previously described [12] and stored at −80 °C until used for assays. DGAT activity was measured in assays with two different acceptors of fatty acids: di-6:0-DAG (only DGAT assays) and *sn*-1-18:1-*sn*-2-[^14^C]18:1-DAG (PDAT and DGAT + PDAT assays). In assays with di-6:0-DAG, 5 nmol [^14^C]acyl-CoA ([^14^C]16:0-CoA, [^14^C]18:1-CoA or [^14^C]22:1-CoA) together with 5 nmol di-6:0-DAG were added with incubation buffer to the microsomal membranes (9 μg of microsomal protein, which was equivalent to approximately 2 nmol of microsomal PtdCho) with incubation buffer (0,05 M HEPES, pH 7.2; 5 mM MgCl_2_; 1 mg BSA/ml) in a final volume 100 μl and incubated for 30 min at 30 °C with shaking (1,250 rpm). In the case of PDAT and DGAT + PDAT assays, [^14^C]18:1-DAG was dissolved in 19 μl of benzene and added to aliquots of microsomal fractions lyophilized overnight (corresponding to 22 μg of microsomal protein). After immediate evaporation of the solvent, buffer (0.05 M HEPES—pH 7.2; 5 mM MgCl_2_, 1 mg BSA/ml) was added and, where measuring combined DGAT + PDAT activities 5 nmol acyl-CoA was included. The assays (final volume 100 μl) were incubated for 30 min at 30 °C with shaking (1,250 rpm). In assays with [^14^C]18:1-DAG + acyl-CoA, formation of [^14^C]TAG was regarded as resulting from both DGAT and PDAT activity. Formation of [^14^C]TAG in assays with only [^14^C]18:1-DAG added was regarded as only PDAT activity. DGAT activity was calculated as amount of [^14^C]TAG in assays with [^14^C]DAG + acyl-CoA minus the amount of [^14^C]TAG in assays with [^14^C]DAG only. However, the standard deviation between triplicate samples in those assays was too high to achieve reliable calculated DGAT activity. Therefore only results from PDAT activity are presented.

At the end of incubation, lipids were extracted from the reaction mixtures into chloroform according to the method of Bligh and Dyer [13] and separated on TLC (silica gel 60 plates; Merck, Darmstadt, Germany) in hexane:diethyl ether:acetic acid (70:30:1 by volume). Radioactive TAG (TAG with two 6:0 moieties clearly separated on TLC from TAG with only long chain fatty acids), products of PDAT and DGAT activity, were visualized and quantified on the plate using electronic autoradiography (Instant Imager, Packard instruments). All assays were repeated independently from three to six times and mean values are presented in the figures.

### Lipid Analysis

Seeds were homogenized in chloroform:methanol:0.15 M acetic acid (1:2:0.8) using a Potter–Elvehjem homogenizer and the lipids were subsequently extracted into chloroform according to Bligh and Dyer [13]. For total lipids analysis, aliquots of the chloroform phase were evaporated and methylated as described below. Individual lipids in the chloroform phase were separated by TLC in hexane:diethyl ether:acetic acid (70:30:1) for neutral lipids or in chloroform:methanol:acetic acid:water (85:15:10:3.5) for separation of polar lipids. Gel, from areas corresponding to the various lipids (identified by means of authentic standards), was removed and lipids were methylated in situ on the gel with 2 % H_2_SO_4_ in dry methanol (60 min at 90 °C). The methyl esters were extracted with hexane and analyzed by GLC equipped with a flame ionization detector and a WCOT fused-silica 50 m × 0.32 mm ID coating CP-Wax 58-CB DF 5 0.2 capillary column (Chrompack International, Middleburg, The Netherlands) with methyl-heptadecanoic acid added as an internal standard.

### Acyl-CoA Analyses

Freshly harvested seeds from different stages of development were frozen in liquid nitrogen and stored at −80 °C until further analyses. The samples were extracted according to Larson and Graham [14], and then analyzed using electrospray ionization tandem mass spectrometry (multi reaction monitoring) or LC–MS/MS MRM in positive ion mode. The LC–MS/MS + MRM analysis (AB4000 QTRAP) followed the methods described by Haynes et al. [15] (Agilent 1200 LC system; Gemini C18 column, 2-mm inner diameter, 150 mm with 5-mm particles). For the purpose of identification and calibration, standard acyl-CoA esters with acyl chain lengths from C14 to C20 were purchased from Sigma as free acids or lithium salts. The experiments were repeated twice independently.

## Results

**Fig. 1:**
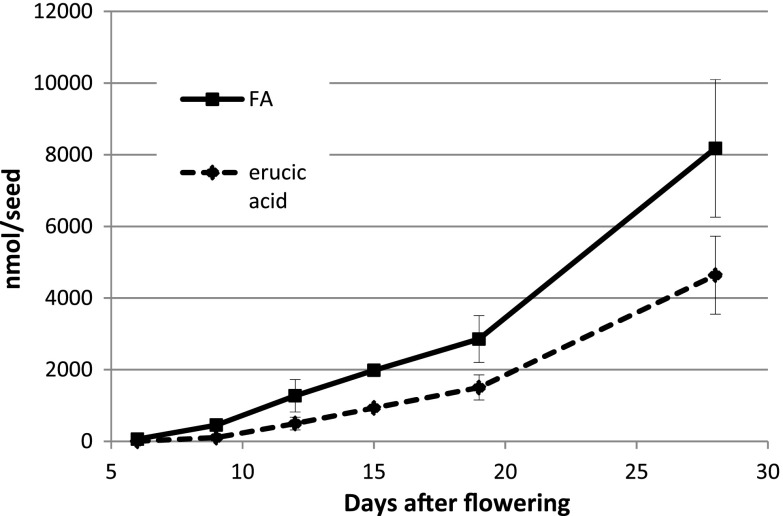
Total fatty acids and erucic acid (nmol/seed) in developing Crambe seeds at different days after flowering (DAF)

**Fig. 2:**
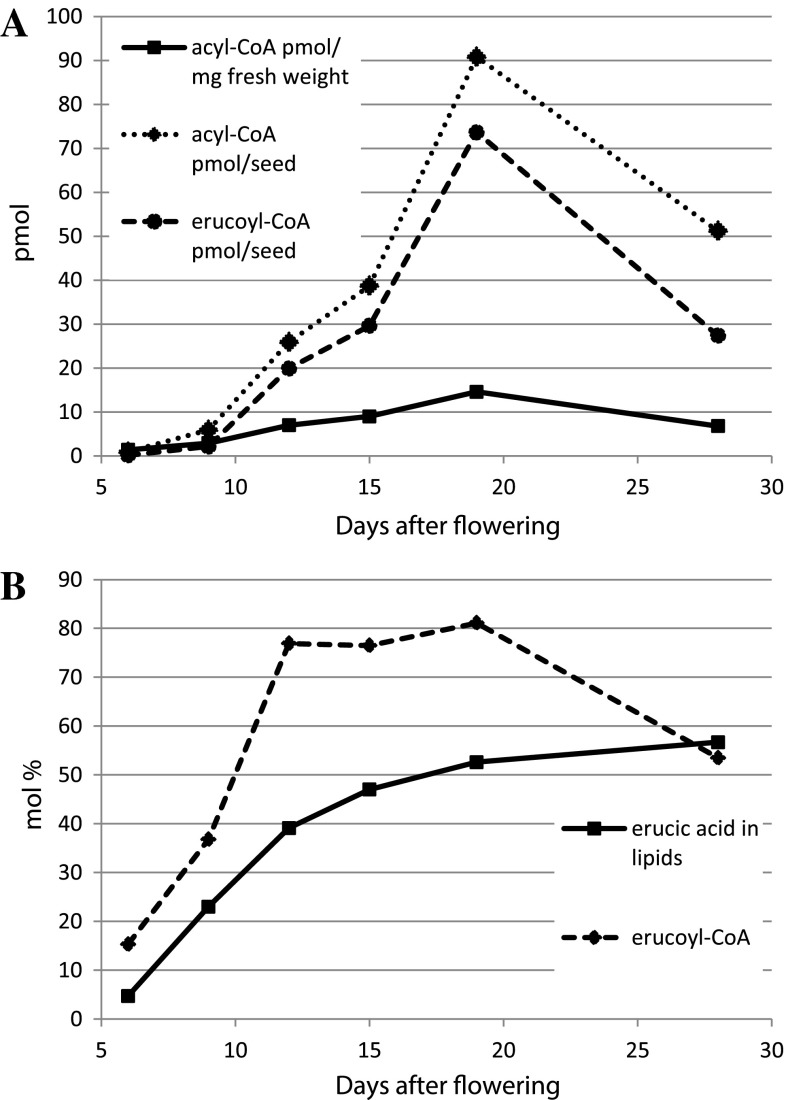
Erucoyl-CoA content in developing Crambe seeds at different days after flowering (DAF). **a** Amount of total acyl-CoA and erucoyl-CoA in Crambe seeds. **b** The percentage of erucoyl groups in acyl-CoA and complex lipids in Crambe seeds

Total fatty acid and 22:1 amounts were measured during Crambe seed (var. Mayer) development (Fig. 1). The total fatty acid and 22:1 acid accumulation were similar in this variety as recently reported in Crambe var. Galactica [10]. During the first 20 DAF, there was a slow phase of lipid accumulation followed by rapid oil accumulation between 20 and 28 DAF. The proportion of 22:1 was very low at 6 DAF (5 mol%) and then gradually increased during seed development to be 57 mol% at 28 DAF. Analysis of total amounts and fatty acid composition in selected individual lipids during seed development are shown in the Supplementary tables (Table S1–S8). Acyl-CoA and 22:1-CoA amount during seed development is presented in Fig. 2a. The acyl-CoA and 22:1-CoA increased both on a per seed basis and per fresh weight up to 20 DAF and showed a substantial decrease at 28 DAF. It should be noted that the seeds had started to enter a desiccation phase at 28 DAF (data not shown). The mol% of 22:1 acid in total lipids and in acyl-CoA is depicted in Fig. 2b. At all stages of seed development except for the last stage, the percentage of 22:1-CoA in the acyl-CoA fraction was much higher than the percentage of 22:1 acid in lipids. For example, at 12 DAF, 22:1-CoA amounted to 77 % of all acyl-CoAs, whereas the lipids contained only 39 mol% of this acid.

**Fig. 3:**
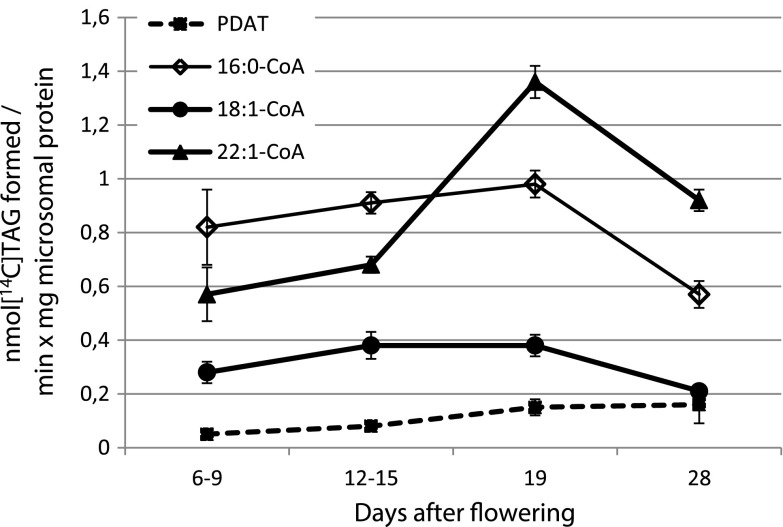
Phospholipid:diacylglycerol acyltransferase (PDAT) and acyl-CoA:diacylglycerol acyltransferase (DGAT) activities in microsomal preparations from developing Crambe seeds at different days after flowering (DAF). DGAT activity was measured with different acyl-CoAs as indicated in the figure

The activity of PDAT was measured by adding radioactive DAG to the microsomal fraction from selected stages of seed development. The specific activity, based on microsomal protein, increased up to 19 DAF (Fig. 3). In a previous report [8] we measured the activity of the DGAT by using the same conditions as in the PDAT assay, but with addition of non-radioactive acyl-CoA. The DGAT activity was then calculated as the amount of TAG formed with acyl-CoA minus the TAG formed without acyl-CoA. However, the high standard deviation seen in the PDAT assays with the Crambe microsomes coupled to the high standard deviation when the assays contained acyl-CoA made an accurate estimation of DGAT activity not possible. We instead measured DGAT activity using di-6:0 DAG, as the acyl acceptor, in combination with radioactive acyl-CoA. Di-6:0-DAG was shown to be an efficient acyl acceptor for both sunflower and safflower DGATs; notably in membranes prepared from those seeds it out-competed acylation of endogenous DAG [8]. In Crambe microsomes, di-6:0-DAG also served as an efficient acyl acceptor for DGAT, but significant proportions of the acyl groups from [^14^C]acyl-CoA was also acylated to endogenous DAG as evident by the formation of radioactive TAG molecules with only long chain acyl groups. The DGAT activity was measured with 16:0-CoA, 18:1-CoA and 22:1-CoA at various stages of seed development. The activities are presented in Fig. 3 as total DGAT activity, i.e., acylation of both endogenous DAG and added di-6:0-DAG. There were considerable differences in specific activity between acyl-CoA species and between time of seed development. DGAT activity was always higher than PDAT activity, ranging from 2.5 to 16 times that activity. There was an over 100 % increase in DGAT activity with 22:1-CoA between membranes prepared from seeds 12–15 DAF and 19 DAF, whereas specific activity with 16:0-CoA and 18:1-CoA were almost not altered between these stages, indicating that expression of a DGAT with high selectivity for 22:1 acid was concomitant with the seed stage when rapid 22:1 acid accumulation commenced.

**Fig. 4:**
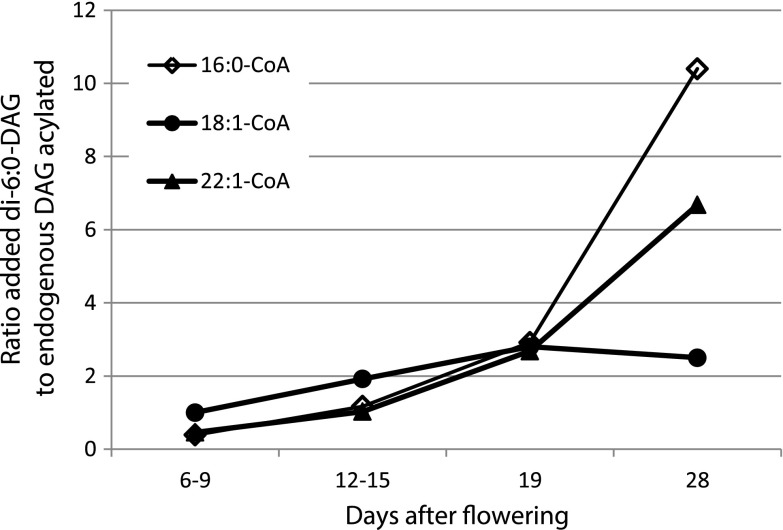
Ratio of specific activity in the acylation of added di-6:0-diacylglyceols (DAG) to endogenous DAG by membrane preparations prepared from Crambe seeds at different days after flowering (DAF). DGAT activity was measured with different acyl-CoAs as indicated in the figure

As mentioned above, Crambe DGAT, in contrast to sunflower and safflower DGATs [8] utilized significant amount of endogenous DAG also in the presence of added di-6:0-DAG. The ratio of utilization of added di-6:0-DAG to endogenous DAG differed considerably between membranes prepared from different stages of development and was dependent less on acyl-CoA species (Fig. 4). At later stages of development, a much higher proportion of added 6:0 DAG than endogenous DAG was acylated with 16:0-CoA and 22:1-CoA than at earlier stages, whereas with 18:1-CoA, this ratio was relatively stable throughout seed development (Fig. 4).

## Discussion

Analyses of the acyl-CoA profile in Crambe seed during seed development revealed that the proportion of 22:1-CoA was much higher than that of the 22:1 acid found in lipids (mainly TAG). This indicates that 22:1-CoA is poorly used by the acylation enzymes compared to other acyl-CoAs. When acyl-CoA profiling was done in developing seed of *Cuphea hookeriana*, which accumulates 25 % of capric acid (10:0) in its oil, the percentage of this fatty acid in the acyl-CoA fraction was significantly lower than found in the TAG, indicating that *Cuphea* acyl transferases have high specificity for this acyl group [16]. On the other hand, transgenic rape seed producing 10:0 fatty acids by expression of *C. hookeriana* medium chain thioesterase had a significantly higher percentage of 10:0 in the acyl-CoA pool than in complex lipids [16], probably due to the poor affinity for 10:0-CoA by the rape seed acyl transferases. Therefore, the amount of 22:1 in Crambe seed TAG might be limited by a poor capacity to acylate this acyl group into the glycerol backbone, thereby causing a build-up of 22:1-CoA and subsequently a product inhibition of the elongation of 18:1-CoA into 22:1-CoA. The amount of 22:1 is very low in the *sn*-2 position of TAG [4] and it has been shown that the Crambe lysosphosphatidic acid acyltransferase (LPAAT), the enzyme responsible for the incorporation of acyl groups at the *sn*-2 position, has very low activity with 22:1-CoA [10]. *Limnanthes douglasii*, which accumulate over 90 % of very long chain fatty acids in its seed TAG, has an LPAAT that has been shown to have good activity with 22:1-CoA [10, 17]. Co-expression of a rape seed elongase (FAE1) and a *L. douglasii* LPAAT in transgenic Crambe seeds led to a significant increase in 22:1 acid in TAG [4]. Expression of the same multigene construct in high 22:1 rape seed did not increase the 22:1 acid in TAG significantly compared to overexpression of FAE1 alone, but rather led to a re-distribution of 22:1 acid between the outer and middle positions of TAG [18]. However, when the transgenic 22:1 rape overexpressing the rape FAE1 and *L. douglasii* LPAT was crossed with a mutant rape seed low in polyunsaturated fatty acids, a substantial increase in the proportion of 22:1 acid in TAG could be observed in the subsequent generations [19]. This indicates that acyltransferase activities for 22:1-CoA was limiting in Crambe whereas in rape, it was the availability of 18:1-CoA substrate for elongation. However, further increase in 22:1 acid in TAG in Crambe could be achieved if conversion of oleic acid to linoleic acid was inhibited by a FAD2-RNAi combined with the expression of FAE1 and *L. douglasii* LPAAT [4], demonstrating that when acyltransferase activity was not limiting, the availability of 18:1-CoA substrate for the FAE became the rate limiting step.

Our assays of DGAT activity in microsomal membranes prepared from seeds at different stages of development suggest that an isoform of DGAT with high specificity for 22:1-CoA is induced at around 19DAF since the specific activity of DGAT with 22:1-CoA at that time point was substantially increased compared to earlier stages of development, whereas the specific activity for 16:0-CoA and 18:1-CoA remained essentially unchanged. This higher activity for 22:1-CoA coincided with a period of rapid TAG and 22:1 acid accumulation.

Despite the fact that the outer positions of TAG in Crambe have over 95 % of very long chain fatty acids [4], significant PDAT activity could be measured in membrane preparations from developing seeds. Since PDAT transfers fatty acids from mainly the *sn*-2 position of PtdCho to the *sn*-3 position of DAG in the formation of TAG [7, 8], it is highly unlikely that PDAT in Crambe transfers any very long chain fatty acids to TAG. No complete stereospecific analysis of Crambe seed TAG has been done, so it is not known how the 5 % of C16 and C18 fatty acids in *sn*-1 + *sn*-3 are distributed between these positions. The specific activities of PDAT in microsomal membranes from Crambe reported here was 50 % of that reported in sunflower membranes and about 20 % of that found in safflower membranes [8]. A similar comparison of the highest DGAT activities (achieved in Crambe with 22:1-CoA as the acyl donor from membranes prepared from seed at 19 DAF) was 20 and 40 % of that found in sunflower and safflower membranes, respectively [8]. Although these comparisons suggest that Crambe PDAT plays a minor role in TAG synthesis compared to both sunflower and safflower, the actual contributions of these enzymes in TAG synthesis might be rather different from the in vitro assays. A caveat for in vitro assays of these enzymes regarding the dilution of added substrates with endogenous substrate is discussed in some detail by Banaś et al. [8]. This point is clearly demonstrated in our work with the different use of added and endogenous DAG substrates in DGAT assays done on Crambe membranes prepared at different developmental stages. Nevertheless, our assays suggest that PDAT could be a significant contributor to the maximal 10 % of C16 and C18 fatty acids that could reside in the *sn*-3 position of TAG in Crambe seeds.

## Electronic supplementary material

Below is the link to the electronic supplementary material.

Supplementary material 1 (DOCX 32 kb)

## Abbreviations

TAG: Triacylglycerol(s)
DAG: Diacylglycerol(s)
PtdCho: Phosphatidylcholine
lysoPtdtOH: Lysophosphatidic acid
PtdOH: Phosphatidic acid
6:0: Hexanoic acid
16:0: Palmitic acid
18:1: 18:1n-9, Oleic acid
18:2: 18:2n-6, Linoleic acid
18:3: 18:3n-3, Alpha-linolenic acid
22:1: 22:1n-9, Erucic acid
GPAT: Acyl-CoA:glycerol-3-phosphate acyltransferase
PDAT: Phospholipid:diacylglycerol acyltransferase
DGAT: Acyl-CoA:diacylglycerol acyltransferase
PDCT: Phosphatidylcholine:diacylglycerol cholinephosphotransferase
LPAAT: Lysophosphatidic acid acyltransferase
FAE: Fatty acid elongase
FAD2: Omega-6-oleate desaturase
RNAi: RNA interference
DAF: Days after flowering
