# Supplementary material for: The Utilization of the Acyl-CoA and the Involvement PDAT and DGAT in the Biosynthesis of Erucic Acid-Rich Triacylglycerols in Crambe Seed Oil
Source: Lipids. 2014 Feb 28;49(4):327–33. doi: 10.1007/s11745-014-3886-7 (PMC3964307; doi:10.1007/s11745-014-3886-7)
Supplement: Supplementary file 1 — Supplementary material 1 (DOCX 32 kb) [file 11745_2014_3886_MOESM1_ESM.docx]

**The utilization of the acyl-CoA and the involvement of phospholipid:diacylglycerol acyltransferase (PDAT) and diacylglycerol acyltransferase (DGAT) in the biosynthesis of erucic acid rich triacylglycerols in *Crambe abyssinica* seed oil**

**Furmanek T, Demski K, Banaś W, Haslam R, Napier J, Stymne S, Banaś A**

**Supplementary tables**

Table S1. Selected parameters characterizing the seeds of *Crambe abyssinica* cv. Mayer at different stages of development.

| Stage of development | Fresh weight  [mg/seed] | Dry weight | | | Lipid contents | | | | | |
| --- | --- | --- | --- | --- | --- | --- | --- | --- | --- | --- |
|  |  | mg/seed | | % of fresh weight | | | nmol FA/seed | | mg/seed | % of dry weight |
| Stage I (6 DAF) | 0.65±0.15 | 0.13 | | 20 | | | 63±27 | | 0.018 | 13.8 |
| Stage II (9 DAF) | 2.01±0.34 | 0.54 | | 27 | | | 455±158 | | 0.134 | 24.8 |
| Stage III (12 DAF) | 3.68±0.39 | 1.29 | | 35 | | | 1274±452 | | 0.376 | 29.1 |
| Stage IV (15 DAF) | 4.3±1.1 | 1.72 | | 40 | | | 1982±70 | | 0.584 | 33.9 |
| Stage V (19 DAF) | 6.2±1.9 | 3.72 | | 60 | | | 2856±655 | | 0.843 | 22.7 |
| Stage VI (28 DAF) | 7.6±1.1 | 6.46 | 85 | | | 8179±1920 | | 2.412 | | 37.3 |

Means ± S.D. shown; 1µmol FA in TAG was calculated as 0.295 mg

The seeds from the lowest part of inflorescence were used for analyses. The seeds were without seed coat. The dry weight is a mean value from several seeds and is presented without SD.

Table S2. The relative content of individual lipid classes in the developing seeds of *Crambe abyssinica*.

| Stage of development | The relative content of fatty acids in individual lipid classes  (% of total fatty acid content) | | | | |
| --- | --- | --- | --- | --- | --- |
|  | Polar lipids | DAG | FA | TAG | SE |
| Stage I | 59.0±11.5 | 10.2±4.3 | 6.4±2.1 | 22.0±6.4 | 2.4±1.1 |
| Stage II | 12.1±1.6 | 2.6±0.3 | 1.3±0.3 | 83.3±1.6 | 0.6±0.3 |
| Stage III | 6.5±0.8 | 1.7±0.3 | 1.3±0.5 | 90.1±1.2 | 0.3±0.1 |
| Stage VI | 2.1±0.3 | 0.9±0.3 | 0.4±0.2 | 96.4±0.5 | 0.2±0.1 |

Means ± S.D. shown

Table S3. Fatty acid composition of total lipids of developing seeds of *Crambe abyssynica* cv. Mayer.

| Stage of  development | fatty acids **(**mol **%)** | | | | | | | |
| --- | --- | --- | --- | --- | --- | --- | --- | --- |
|  | 16:0 | 18:0 | 18:1 | 18:2 | 18:3 | 20:1 | 22:1 | other |
| StageI | 13.0±2.5 | 5.6±1.3 | 24.9±2.9 | 19.9±2.5 | 19.5±4.1 | 4.4±1.4 | 4.7±1.8 | 7.9±2.0 |
| StageII | 6.9±1.9 | 3.2±1.1 | 21.7±2.7 | 16.3±1.1 | 13.0±1.1 | 7.8±0.5 | 23.0±5.9 | 8.1±1.0 |
| StageIII | 4.6±0.7 | 1.9±0.3 | 15.3±0.8 | 13.4±1.4 | 10.5±1.8 | 6.8±0.5 | 39.1±2.9 | 8.4±1.7 |
| StageIV | 3.7±0.7 | 1.3±0.3 | 12.6±1.3 | 11.6±1.0 | 10.6±1.2 | 4.3±1.2 | 47.0±2.5 | 9.6±1.8 |
| StageV | 3.0±0.4 | 0.8±0.2 | 12.6±1.3 | 10.4±1.2 | 9.0±2.0 | 2.1±0.7 | 52.6±2.2 | 9.6±1.5 |
| StageVI | 1.7±0.2 | 0.5±0.1 | 12.6±0.8 | 9.3±0.7 | 7.8±0.3 | 1.2±0.3 | 56.7±0.7 | 10.0±0.8 |

Means ± S.D. shown

Table S4. Content and composition of acyl-CoA in developing seeds of *Crambe abyssynica* cv. Mayer.

| Stage of develop-ment | pmol/mg  fresh weight | pmol/seed | Acyl-CoA **(%)** | | | | | | | |
| --- | --- | --- | --- | --- | --- | --- | --- | --- | --- | --- |
|  |  |  | **16:0** | **18:0** | **18:1** | **18:2** | **18:3** | **20:1** | **22:1** | **other** |
| Stage I | **1.4** | 0.9 | 10.5 | 5.3 | 18.9 | 10.6 | 7.7 | 8.4 | **15.3** | 23.3 |
| Stage II | **2.9** | 5.9 | 5.1 | 2.9 | 16.7 | 4.7 | 2.5 | 12.0 | **36.8** | 18.3 |
| Stage III | **7.0** | 25.9 | 1.9 | 0.4 | 9.0 | 1.9 | 1.3 | 1.5 | **76.9** | 7.1 |
| Stage IV | **9.0** | 38.7 | 1.5 | 1.1 | 6.8 | 1.5 | 0.9 | 6.9 | **76.5** | 4.8 |
| Stage V | **14.6** | 90.8 | 1.1 | 0.9 | 5.5 | 1.0 | 0.6 | 5.3 | **81.1** | 4.5 |
| Stage VI | **6.8** | 51.2 | 4.3 | 2.1 | 13.5 | 3.5 | 0.8 | 16.1 | **53.5** | 6.2 |

Means of duplicate shown

Table S5. Fatty acid composition of individual lipid classes of *Crambe abyssynica* cv Mayer seeds at the early stage of development (stage II).

| Lipid  class | fattyacids**(**mol **%)** | | | | | | | |
| --- | --- | --- | --- | --- | --- | --- | --- | --- |
|  | **16:0** | **18:0** | **18:1** | **18:2** | **18:3** | **20:1** | **22:1** | **other** |
| **Polar** | 13.5±0.6 | 3.0±0.3 | 11.1±1.1 | 26.7±2.0 | 35.4±2.0 | 2.0±0.3 | 1.2±0.3 | 7.1±0.3 |
| **DAG** | 11.1±3.5 | 4.5±2.0 | 23.1±2.2 | 22.7±2.8 | 15.2±3.2 | 5.9±0.8 | 10.9±2.1 | 6.5±1.3 |
| **FA** | 11.7±4.7 | 9.9±4.7 | 21.6±2.4 | 13.0±2.3 | 7.4±1.0 | 7.5±1.7 | 25.2±6.0 | 3.7±2.5 |
| TAG | 5.3±0.5 | 2.7±0.2 | 23.1±2.5 | 15.0±0.6 | 9.8±0.6 | 8.6±0.5 | 27.4±3.8 | 8.1±0.5 |
| SE | 12.3±8.3 | 5.3±4.5 | 8.0±1.1 | 40.1±8.1 | 23.0±4.4 | n.d. | 1.8±1.7 | 9.5±1.9 |

Means ± S.D. shown; n.d. = not detected

Table S6. Fatty acid composition of individual lipid classes of *Crambe abyssynica* cv Mayer seeds at the late stage of development (stage VI).

| Lipid  class | Fatty acids **(**mol**%)** | | | | | | | |
| --- | --- | --- | --- | --- | --- | --- | --- | --- |
|  | **16:0** | **18:0** | **18:1** | **18:2** | **18:3** | **20:1** | **22:1** | **other** |
| **Polar** | 10.5±2.8 | 2.1±1.0 | 36.0±4.3 | 29.7±2.8 | 10.4±3.2 | 1.9±0.3 | 5.4±1.1 | 4.0±2.2 |
| **DAG** | 5.0±1.5 | 2.4±0.5 | 22.1±2.8 | 12.1±2.8 | 7.5±1.1 | 1.6±0.5 | 46.4±7.6 | 2.5±2.5 |
| **FA** | 11.7±6.6 | 9.0±6.1 | 12.1±3.2 | 8.5±1.9 | 5.4±1.3 | 1.0±0.5 | 48.0±7.8 | 4.3±3.3 |
| TAG | 1.4±0.2 | 0.5±0.1 | 12.6±0.9 | 8.5±0.5 | 7.2±0.2 | 1.1±0.2 | 57.9±0.9 | 10.8±1.0 |
| SE | 20.3±9.5 | 12.8±8.4 | 14.3±8.4 | 22.8±8.3 | 9.8±4.7 | 4.7±2.8 | 11.9±9.8 | 8.2±4.2 |

Means ± S.D. shown

Table S7. Polar lipid classes of *Crambe abyssynica* cv Mayer seeds at the early stage of development (stage II).

| Lipid  class | % of total polar lipids | Fatty acids **(**mol **%)** | | | | | | | |
| --- | --- | --- | --- | --- | --- | --- | --- | --- | --- |
|  |  | **16:0** | **18:0** | **18:1** | **18:2** | **18:3** | **20:1** | **22:1** | **other** |
| **PI+LPE** | **4.7±2.4** | 43.3±3.3 | 3.7±0.6 | 18.7±1.9 | 25.3±0.4 | 7.8±1.0 | n.d. | n.d. | 1.2±0.6 |
| **PS** | **1.5±0.1** | 40.0±2.3 | 8.9±0.4 | 12.5±0.6 | 20.8±5.7 | 13.8±0.4 | n.d. | n.d. | 4.0±2.0 |
| **PC** | **38.8±1.6** | 13.6±0.1 | 1.9±0.2 | 26.5±1.4 | 32.5±1.5 | 16.4±3.3 | 2.8±1.1 | 2.3±1.1 | 4.0±1.1 |
| **DGDG** | **6.2±0.8** | 6.3±0.3 | 4.3±0.6 | 1.7±0.1 | 8.3±0.2 | 74.8±0.9 | n.d. | n.d. | 4.6±0.9 |
| **PG** | **3.7±1.4** | 61.2±3.8 | 2.3±0.1 | 10.3±0.4 | 17.9±0.5 | 7.5±2.2 | n.d. | n.d. | 0.8±0.4 |
| **PE** | **14.8±0.9** | 20.4±0.5 | 1.7±0.3 | 15.2±0.2 | 38.3±4.9 | 17.1±5.2 | 1.6±0.1 | 1.9±0.8 | 3.8±1.6 |
| **PA** | **1.5±0.1** | 15.0±1.1 | 3.0±0.6 | 21.7±0.3 | 34.7±2.3 | 22.2±1.5 | n.d. | n.d. | 3.4±0.8 |
| **X** | **14.4±2.8** | 17.2±1.2 | 1.7±0.3 | 25.5±1.3 | 35.3±3.9 | 12.0±1.3 | 2.5±0.8 | 2.7±0.5 | 3.1±1.3 |
| **MGDG** | **12.5±1.6** | 3.8±1.2 | 0.8±0.4 | 3.4±0.4 | 13.1±3.2 | 51.9±3.0 | n.d. | n.d | 27.4±2.6  (22.9±1.4) |
| **Rest** | **1.9±0.3** | 44.1±6.4 | 14.5±2.4 | 22.1±3.1 | 12.3±6.0 | 3.9±1.5 | n.d. | n.d. | 3.1±0.9 |

Means ± S.D. shown; n.d. = not detected; MGDG “others” – in brackets 16:3

X – lipid localised between PA and MGDG;

Table S8. Polar lipid classes of *Crambe abyssynica* cv Mayer seeds at the late stage of development (stage VI).

| Lipid  class | % of total polar lipids | fattyacids**(**mol **%)** | | | | | | | |
| --- | --- | --- | --- | --- | --- | --- | --- | --- | --- |
|  |  | **16:0** | **18:0** | **18:1** | **18:2** | **18:3** | **20:1** | **22:1** | **other** |
| **PI+LPE** | **7.3±2.7** | 47.4±2.7 | 4.5±0.5 | 14.1±2.4 | 23.0±2.7 | 8.8±2.3 | n.d. | 1.2±0.2 | 1.0±0.3 |
| **PC** | **56.1±2.2** | 12.0±1.5 | 2.5±0.9 | 40.3±5.8 | 24.4±5.1 | 9.0±2.8 | 2.1±0.4 | 4.2±0.3 | 5.4±1.2 |
| **PG** | **2.7±0.4** | 57.9±3.6 | 5.8±1.2 | 10.3±1.8 | 19.5±2.9 | 5.6±0.5 | n.d. | n.d. | 0.9±0.3 |
| **PE** | **24.7±1.4** | 21.3±5.8 | 2.6±1.0 | 17.6±3.4 | 35.2±5.9 | 11.0±2.4 | 1.3±0.1 | 6.0±0.4 | 5.0±1.3 |
| **PA** | **1.4±0.1** | 19.8±2.6 | 8.0±1.2 | 21.6±1.4 | 30.7±1.6 | 11.8±1.7 | 0.7±0.5 | 7.0±0.7 | 0.4±0.2 |
| **Rest** | **7.8±1.4** | 39.7±7.3 | 20.6±6.4 | 12.5±3.0 | 13.6±2.2 | 7.7±3.2 | trace | 4.8±1.2 | 1.1±0.4 |

Means ± S.D. shown; n.d. = not detected;
